# Subcapsular splenic hematoma and spontaneous hemoperitoneum in a cocaine user

**DOI:** 10.1590/0100-3984.2015.0203

**Published:** 2017

**Authors:** Bruno Niemeyer de Freitas Ribeiro, Rafael Santos Correia, Tiago Medina Salata, Fernanda Salata Antunes, Edson Marchiori

**Affiliations:** 1 Instituto Estadual do Cérebro Paulo Niemeyer, Rio de Janeiro, RJ, Brazil; 2 Hospital Casa de Portugal / Clínica 3D Diagnóstico por Imagem, Rio de Janeiro, RJ, Brazil; 3 Universidade Federal do Rio de Janeiro (UFRJ), Rio de Janeiro, RJ, Brazil

*Dear Editor*,

A 23-year-old male patient presented with a 36-h history of intense, sudden, progressive abdominal pain, predominantly in the left hypochondrium, irradiating to the ipsilateral infrascapular region. He reported no previous trauma, fever, headache, fatigue, myalgia, arthralgia, skin alterations, or comorbidities. During the clinical interview, he reported moderate smoking and the routine use of an illicit drug (cocaine), including hours prior to the onset of pain. On physical examination, he was well-oriented, hemodynamically stable, and afebrile. The serology was negative for hepatitis B, hepatitis C, and dengue, and the results were normal for antineutrophil cytoplasmic antibody, antinuclear factor, the venereal disease research laboratory test, urea, creatinine, erythrocyte sedimentation rate, C-reactive protein, and coagulation profile. Hemoglobin electrophoresis showed no alterations.

**Figure 1:**
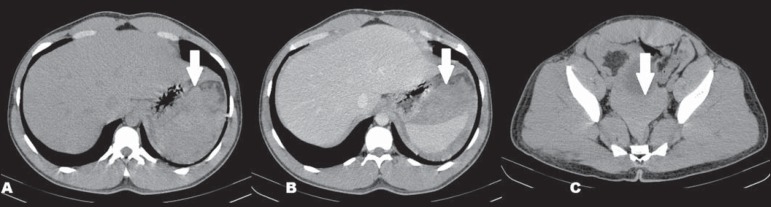
CT scan showing subcapsular hematoma and hemoperitoneum. **A**: CT, axial slice, without contrast, demonstrating dense collections (arrow) adjacent to the spleen. **B**: Contrast-enhanced axial CT, showing the dense collections adjacent to the spleen (arrow), without contrast enhancement, indicative of subcapsular hematoma. **C**: CT, axial slice, without contrast, demonstrating spontaneously dense free liquid in the pelvis (arrow), indicative of hemoperitoneum.

Computed tomography (CT) showed a dense collection, compatible with hematic material, in close proximity to the spleen, as well as showing hemoperitoneum (Figure 1). Arteriography showed no abnormalities. Exploratory laparotomy revealed subcapsular splenic hematoma and confirmed the hemoperitoneum, with no evidence of a lesion within the cavity.

Given that there was no perisplenic trauma or adhesions suggestive of previous trauma and that the macroscopic aspect of the spleen was normal on the CT scan and in the exploratory laparotomy, together with the facts that diseases affecting the splenic parenchyma were ruled out and that the patient had used cocaine immediately prior to the episode, we established the working diagnosis of nontraumatic splenic hemorrhage secondary to cocaine use. During clinical follow-up, the patient progressed well, without complications.

Recent studies in the radiology literature of Brazil have emphasized the importance of CT and magnetic resonance imaging scans to improving the diagnosis in nontraumatic abdominal disorders^(1-5)^. Splenic hemorrhages are rarely encountered without prior trauma and can have fatal consequences, which makes their early diagnosis essential. The main nontraumatic conditions include neoplasms, as well as inflammatory/infectious, iatrogenic, and mechanical processes^(6)^.

The clinical signs of nontraumatic splenic hemorrhage are similar to those found in cases resulting from trauma, including pain in the upper left quadrant, with or without irradiation to the left shoulder, caused by diaphragmatic irritation, evolving to hemodynamic instability in the most severe cases. Such manifestations are nonspecific and cannot be characterized solely by physical examination. Therefore, in hemodynamically stable patients, CT evaluation is fundamental to the characterization of the affected organs^(6,7)^.

Currently, Brazil is the second largest consumer of cocaine and its derivatives, the leader being the United States^(8)^. The mechanism thought to trigger bleeding during or after cocaine use is stimulation of alpha-adrenergic receptors, which produce vasoconstriction with a consequent increase in abdominal blood pressure and a reduction of up to 20% of the splenic volume, promoting high-pressure blood flow in a retracted parenchyma and with a low concentration of connective tissue, making the spleen more prone to bleeding, which can be triggered even by coughing^(6,9)^.

In nontraumatic splenic hemorrhage, the differential diagnoses include dengue, infectious mononucleosis, polyarteritis nodosa, segmental arterial mediolysis, neoplasms, coagulopathy. and hemoglobinopathy^(6,7,9-11)^.

In conclusion, although nontraumatic splenic hemorrhage is uncommon, the possibility of cocaine use as a triggering event should be considered, especially in young, previously healthy patients with no comorbidities to explain such an event.
